# Synchronous motor imagery and visual feedback of finger movement elicit the moving rubber hand illusion, at least in illusion-susceptible individuals

**DOI:** 10.1007/s00221-023-06586-w

**Published:** 2023-03-16

**Authors:** Christopher C. Berger, Sara Coppi, H. Henrik Ehrsson

**Affiliations:** 1 Department of Neuroscience, Karolinska Institutet, Stockholm, Sweden; 2 Division of Biology and Biological Engineering/Computation and Neural Systems, California Institute of Technology, Pasadena, CA USA

**Keywords:** Motor imagery, Body ownership, Sense of agency, Multisensory plasticity

## Abstract

Recent evidence suggests that imagined auditory and visual sensory stimuli can be integrated with real sensory information from a different sensory modality to change the perception of external events via cross-modal multisensory integration mechanisms. Here, we explored whether imagined voluntary movements can integrate visual and proprioceptive cues to change how we perceive our own limbs in space. Participants viewed a robotic hand wearing a glove repetitively moving its right index finger up and down at a frequency of 1 Hz, while they imagined executing the corresponding movements synchronously or asynchronously (kinesthetic-motor imagery); electromyography (EMG) from the participants’ right index flexor muscle confirmed that the participants kept their hand relaxed while imagining the movements. The questionnaire results revealed that the synchronously imagined movements elicited illusory ownership and a sense of agency over the moving robotic hand—the moving rubber hand illusion—compared with asynchronously imagined movements; individuals who affirmed experiencing the illusion with real synchronous movement also did so with synchronous imagined movements. The results from a proprioceptive drift task further demonstrated a shift in the perceived location of the participants’ real hand toward the robotic hand in the synchronous versus the asynchronous motor imagery condition. These results suggest that kinesthetic motor imagery can be used to replace veridical congruent somatosensory feedback from a moving finger in the moving rubber hand illusion to trigger illusory body ownership and agency, but only if the temporal congruence rule of the illusion is obeyed. This observation extends previous studies on the integration of mental imagery and sensory perception to the case of multisensory bodily awareness, which has potentially important implications for research into embodiment of brain–computer interface controlled robotic prostheses and computer-generated limbs in virtual reality.

**Supplementary Information:**

The online version contains supplementary material available at 10.1007/s00221-023-06586-w.

## Introduction

How do we sense that our body is our own and that we are the agent of our actions? Research in psychology and cognitive neuroscience on the feeling of body ownership suggests that the experience of ‘this is my body’ (i.e., body ownership) is the result of the integration of sensory signals from our different senses (Blanke et al. 2015; Ehrsson 2012) and that the feeling ‘these are my actions’ (i.e., the sense of agency) is the result of the agreement between our intentions (Haggard 2017) to act and the sensory consequences of our actions (Engbert et al. 2007; Haggard 2017; Kristjánsson et al. 2016; Lynn et al. 2010; Moore and Obhi 2012) as well as the match between the predicted sensory consequences of movement and their precise sensory feedback (Frith et al. 2000). Herein, we asked the following: what if some of those sensorimotor signals were not real but imagined instead?

Mental imagery refers to the human ability to mentally simulate sensory experiences at will. Dominating perceptually based theories of mental imagery (Kosslyn et al. 1978, 1993, 2001) state that mental imagery engages some of the same cognitive processes (Farah 1985; Schlegel et al. 2013) and neural mechanisms (Berger and Ehrsson 2014; Ehrsson et al. 2003; Kosslyn et al. 1993, 2001; Pearson et al. 2015) as actual perception (Pearson 2019; Pearson and Kosslyn 2013). Mental imagery is thus similar to veridical perception, although less vivid, and typically clearly separated from the latter in our minds (Koenig-Robert and Pearson 2021; Pearson 2019). However, previous studies on mental imagery and cross-modal perceptual illusions have shown that the boundary between imagination and sensation is not as fixed as is perhaps often assumed (Berger and Ehrsson 2013, 2014, 2017, 2018; Lacey et al. 2010). Berger and colleagues found that sensory signals in one sensory modality can integrate with signals generated centrally by mental imagery in another modality and trigger multisensory illusions, but, critically, only if the contents, timing, and spatial characteristics of mental imagery obey the same perceptual rules that govern the veridical illusion in question. Multisensory illusions require that the sensory signals in different sensory modalities obey temporal, spatial and other congruence rules (Sekuler et al. 1997; Shams et al. 2000; Thurlow and Jack 1973), so that, for example, a sound (e.g., a dog’s bark) and visual stimuli (e.g., the sight of a barking dog) are perceptually bound into a coherent multisensory experience (of a single barking dog) only if they originate sufficiently close in time and space and if they match in terms of information content (e.g., a dog stimulus and a cat stimulus fuse less effectively) (Stein and Stanford 2008). Specifically, Berger and colleagues showed that several classic audiovisual illusions, such as the cross-bounce illusion (Berger and Ehrsson 2013, 2017), the McGurk illusion (Berger and Ehrsson 2013), and the ventriloquist illusion (Berger and Ehrsson 2013, 2016), could be triggered by replacing visual or auditory stimuli with visual and auditory imagery of the corresponding stimulus, respectively, according to the precise perceptual rules of the illusion. This indicates that mental imagery in one modality can integrate with actual sensory signals in a different sensory modality and elicit multisensory perception that is part based on reality and part based on imagination. Moreover, repeated integration of visual imagery and auditory stimulation triggering the ventriloquist illusion leads to a ventriloquism aftereffect (just as after the veridical illusion), which suggests that the imagery-induced ventriloquist illusion leads to short-term cross-modal plasticity and multisensory recalibration in central audiovisual representations (Berger and Ehrsson 2018). Furthermore, an fMRI study found that the imagery-induced ventriloquist illusion was associated with increased activity in a section of the superior temporal cortex that is associated with audiovisual integration and with increased functional connectivity between this superior temporal area and the auditory cortex (Berger and Ehrsson 2014). Collectively, these studies indicate that mental imagery of auditory and visual events is associated with the activation of sufficiently strong and perception-like central processes and neural representations capable of integrating with veridical sensory signals and triggering multisensory perceptual illusions. However, these previous studies all investigated the perception of external audiovisual events; thus, it is unclear whether the same principle holds true for other modalities and, in particular, whether mental imagery of bodily sensations can trigger multisensory bodily illusions and cause changes in the sense of bodily self, arguably the most basic form of self-representation (Blanke et al. 2015; De Vignemont 2018; Ehrsson 2007; Gallagher 2000; Tacikowski et al. 2020; Tsakiris 2017).

A classic paradigm to investigate the sense of body ownership and body representation in healthy individuals is the rubber hand illusion (Botvinick and Cohen 1998). The rubber hand illusion is a multisensory bodily illusion (Ehrsson 2020) where people perceive somatosensory sensations originating from a rubber hand in full view coupled with a bodily feeling that the false hand is their own and part of their body (Botvinick and Cohen 1998; Ehrsson 2012; Longo et al. 2008). The classic method to induce this illusion is to apply synchronous brushstrokes to the rubber hand and the real hand, which is hidden behind a screen; after a brief period of repeated stroking (10 to 12 s in most cases), the illusion is induced (Chancel et al. 2022; Ehrsson et al. 2004; Lloyd 2007). Critically, the illusion depends on spatial, temporal, and other multisensory constraints in line with multisensory integration principles (Ehrsson 2012, 2020; Ehrsson et al. 2004). For example, the seen and felt brushstrokes must be sufficiently synchronous, and asynchronies of more than 300 ms break the illusion (and even shorter delays can reduce the illusion strength; Chancel et al. 2022; Chancel and Ehrsson 2020) in line with the temporal principle of multisensory integration (Stein and Stanford 2008). Similarly, the spatial orientation of the rubber hand must resemble the orientation of the hidden real hand, and large spatial discrepancies break the illusion (Ehrsson et al. 2004; Fang et al. 2019; Ide 2013; Tsakiris and Haggard 2005) in line with the spatial principle of multisensory integration. Thus, the illusory perception of the rubber hand as one’s own comes about from the binding of the visual impressions from the rubber hand and the somatosensory sensations from the hidden real hand into a coherent multisensory representation of the rubber hand as part of one’s body (Blanke et al. 2015; Ehrsson 2012, 2020; Ehrsson et al. 2004; Kilteni et al. 2015). In more recent probabilistic models of body ownership, the rubber hand illusion is not conceptualized as being determined by fixed multisensory rules and constraints but rather arising as the outcome of an automatic perceptual decision process where the brain’s perceptual system infers how likely it is for the different sensory signals to come from a common cause given the spatial proximity, simultaneity and temporal correlation of the sensory signals; their relative uncertainty; and prior constraints extracted from the environment and previous experience (Chancel et al. 2022; Fang et al. 2019; Kilteni et al. 2015; Körding et al. 2007; Samad et al. 2015; Sato et al. 2007).

Of particular relevance to the current study is the finding that the rubber hand illusion can also be elicited with synchronous finger movements instead of brush stroking: the ‘moving rubber hand illusion’ (Kalckert and Ehrsson 2012). Kalckert and Ehrsson (2012) found that synchrony between the felt movements of one’s hidden real index finger and the seen finger movements of a wooden model hand wearing a rubber glove elicited subjective feelings of rubber hand ownership coupled with a significant drift in the perceived location of one’s real hand (hidden from view) toward the seen rubber hand, which is characteristic of successful rubber hand illusion induction (‘proprioceptive drift; Botvinick and Cohen 1998; Tsakiris and Haggard 2005). Asynchronous seen and felt movements abolish the illusion according to temporal congruence principles similar to those of the classic rubber hand illusion (Dummer et al. 2009; Ismail and Shimada 2016; Kalckert and Ehrsson 2012), as does introducing a substantial spatial incongruence between the orientations of the rubber hand and the real hand (Abdulkarim et al. 2023; Kalckert and Ehrsson 2012, 2014). Thus, illusory body ownership of the moving rubber hand depends on multisensory integration of visual, kinesthetic and proprioceptive signals. An important difference between the moving rubber hand illusion and the classic illusion is that when the illusion is induced by active movement, the participant also experiences a sense of agency over the rubber hand’s finger movement, i.e., the cognitive feeling that one is in voluntary control of the action one observes (Haggard 2017) (as mentioned above). This sense of agency over the rubber hand’s movements arises because the motor intentions associated with voluntarily generating finger movements match the expected sensory consequences of the voluntary movement in terms of visual feedback of the rubber hand’s movements (Kalckert and Ehrsson 2012).

Therefore, can mental imagery be used to trigger the rubber hand illusion and cause changes in the sense of body ownership? This question is interesting not only from a basic science perspective, in the sense of advancing our understanding of the relationship between mental imagery and multisensory perception of bodily self, but also from an applied neuroscience perspective. A major goal in brain–computer interface (BCI) and neurorehabilitation research is to develop advanced prosthetic, robotic, and computer-generated virtual limbs that can be controlled by people with paralyzed or amputated limbs by registering brain signals (e.g., with electroencephalography, EEG; (Hochberg et al. 2006, 2012; McFarland and Wolpaw 2017; Murphy et al. 2017). It is unclear to what extent such prosthetic and virtual limbs can be embodied and experienced as real limbs, although encouraging results indicate that a sense of agency can be evoked (Nierula et al. 2021; Serino et al. 2022). Motor imagery (Jeannerod and Decety 1995)—imaging a movement without actually executing it—has often been used to investigate possible changes in body ownership and the feeling of agency over virtual and robotic limbs in various BCI setups involving healthy volunteers with intact limbs (Batula et al. 2017; Pfurtscheller and Neuper 2001). Motor imagery is a form of internal simulation of action that can be used in motor preparation and mental rehearsal to improve action execution (Page et al. 2009; Witt and Proffitt 2008). This type of imagery engages similar motor programs (Jeannerod and Decety 1995; Sirigu et al. 1996), central perceptual representations (Naito and Sadato 2003), and neural representations (Decety 1996; Ehrsson et al. 2003; Hétu et al. 2013; Pelgrims et al. 2011) as real movement execution. In BCI experiments using motor imagery, the participants are typically instructed to imagine different movements (e.g., opening and closing a fist or moving the left or right hand), which, through the BCI, is translated to corresponding movements of the prosthesis or virtual limb; such BCI setups are thus similar to the moving rubber hand illusion paradigm but without somatosensory feedback of the movements. One study reported how motor imagery elicits a sense of agency over a BCI-controlled virtual arm (Perez-Marcos et al. 2009), but because the virtual hand was controlled by motor imagery of the foot, not the hand, the rubber hand illusion was probably not elicited, as such anatomical incongruence (Guterstam et al. 2011) would have prevented the experience of ownership over the virtual limb (this view is further supported by the lack of significant proprioceptive drift in (Perez-Marcos et al. 2009). Alimardani and coworkers (Alimardani et al. 2013) reported the embodiment of humanoid robotic hands controlled by a BCI, but the subjective ratings mixed agency and ownership experiences, and the reported effect was maintained even with long and noticeable delays (≈1 s) in the BCI system (see also Alimardani et al. 2016). This finding is odd because asynchronies longer than approximately 300 ms significantly disrupt the moving rubber hand illusion (Ismail and Shimada 2016; Shimada et al. 2009), and a one-second delay should break it (Kalckert and Ehrsson 2012), as discussed above. Finally, Braun and colleagues (Braun et al. 2016) presented encouraging results showing that motor imagery of opening and closing the first combined with congruent visual feedback of a robotic hand performing the same action led to significant affirmative ratings of ownership and agency compared to a control condition when the robotic hand was presented in a spatially incongruent orientation (rotated 180 degrees). However, in this study, no asynchronous control condition was included, and the proprioceptive drift measure of the rubber hand illusion was not used. Two further limitations of the previous work are relevant to mention here. First, the type of motor imagery (e.g., kinesthetic, visual, or mixed) and the perspective (first- or third-person perspective) were not described in the majority of the studies (Alimardani et al. 2013, 2016; Perez-Marcos et al. 2009), which is a general problem in motor imagery research (Van Caenegem et al. 2022). Thus, it is unclear precisely how the participants performed the motor imagery in this study and whether it involved kinesthetic-motor imagery from the first-person perspective as would be required for triggering the moving rubber hand illusion according to this illusion’s multisensory rules or visual imagery of action, which would not. Furthermore, in all studies mentioned above except for the one by Braun et al. (2016), muscular activity in the real hand was not monitored to exclude possible tiny movements or static muscular contractions that can occur spontaneously when people engage in vivid motor imagery (Guillot et al. 2007), which would provide afferent somatosensory feedback from the real limb that could drive an ownership illusion rather than mental imagery per se. Thus, it is not clear to what extent illusory body ownership can be elicited by motor imagery in BCI setups, and to the best of our knowledge, no study has investigated whether motor imagery can be used to elicit the classic moving rubber hand illusion without a BCI or virtual reality technology; this gap in the literature is a problem because more technologically complex paradigms assume that this should be possible. Hence, we reasoned that what is lacking is a simple “low-tech” study that demonstrates that the moving rubber hand illusion can be elicited by motor-kinesthetic imagery according to the multisensory rules of the illusion in the absence of small finger movements or muscular contractions.

Thus, in this study, we examined the question of whether motor imagery can be used to elicit the moving rubber hand illusion by replacing actual somatosensory feedback from the hidden real hand according to the temporal rule of the illusion. As mentioned, we used ‘kinesthetic-motor imagery’ (Jeannerod and Decety 1995), which is motor imagery where the person is imagining the sensation of doing the action from the first-person perspective. This type of motor imagery is intimately linked to kinesthetic imagery (Naito and Sadato 2003), which, in principle, could allow for a spatiotemporal match between the content and timing of mental kinesthetic images and the veridical visual feedback of the moving rubber hand that is critical for eliciting multisensory illusions with mental imagery in our theory (as described above). Thus, we stressed to the participants to imagine the somatosensory feeling associated with generating repetitive index finger movement of their hidden real hand (kinesthetic-motor imagery from the first-person perspective) while a robotic hand was moving its index finger either synchronously or asynchronously during 1-min periods in full view. Both a subjective measure (questionnaire ratings) and a more objective indirect behavioral measure (the perceived location of the participant’s real hand, i.e., proprioceptive drift; Abdulkarim and Ehrsson 2016; Botvinick and Cohen 1998; Tsakiris and Haggard 2005) were collected in line with a large number of previous rubber hand illusion studies (e.g., Caspar et al. 2015; Kalckert and Ehrsson 2012, 2014; Tsakiris et al. 2010) to examine the hypothesis that synchronously imagined movements—but not asynchronous imagined movements—would trigger the moving rubber hand illusion both at the subjective level and at the behavioral level (proprioceptive drift). Moreover, electromyography (EMG) was used to rule out any weak muscular contractions in muscles controlling the index finger, ensuring that the hypothesized effects were driven by motor imagery and not actual sensory feedback from the real index finger. We also hypothesized that synchronous imagined and real finger movements would trigger a feeling of agency over the seen robotic hand movements due to the match between internally simulated motor intentions and matching expected sensory feedback (Engbert et al. 2007; Frith et al. 2000), which would be in line with earlier imagery-controlled BCI studies (Braun et al. 2016; Nierula et al. 2021; Serino et al. 2022).

## Materials and methods

### Participants

Twenty-four participants participated in the experiment (mean age = 26.21 years, SD = 5.59; 14 females). Two additional participants were also recruited to participate but did not complete the experiment due to failure to follow instructions. The data for these latter two participants were not analyzed and were not included in the results. We conducted two power analyses based on two previous studies using similar independent and dependent measures (Kalckert and Ehrsson 2012, 2014) to detect the minimum sample size. We used RStudio software (R Core Team 2021) and the functions pwr.t.test() and pwr.r.test() from the “pwr” package (Champely 2020).

The first power analysis was based on Kalckert and Ehrsson (2012). In their questionnaire data (Experiment 1), the comparison between Agency Sync and Agency Async conditions showed a Cohen’s *d*_z_ of 1.55 and a power of 1 − *β* = 0.99. Therefore, to obtain a similar effect size with a power of 1 − *β* = 0.95, the minimum sample size would be 8 subjects. In their proprioceptive drift data (Experiment 2), the comparison between Agency Sync and Agency Async conditions showed a Cohen’s *d*_z_ of 0.846 and a power of 1 − *β* = 0.948. Therefore, to obtain a similar effect size with a power of 1 − *β* = 0.95, the minimum sample size would be 20 subjects.

The second power analysis was based on Kalckert and Ehrsson (2014). In the questionnaire data, the comparison between Agency Sync and Agency Async conditions led to a correlation coefficient (*r*_C_) of 0.831 and a power of 1 − *β* = 1. Therefore, to obtain a similar effect size with a power of 1 − *β* = 0.95, the minimum sample size would be 12 subjects. In this study’s proprioceptive drift data, the comparison between Agency Sync and Agency Async conditions showed a Cohen’s *d*_z_ of 0.762 and a power of 1 − *β* = 0.898. Therefore, to obtain a similar effect size with a power of 1 − *β* = 0.95, the minimum sample size would be 24 subjects.

Three considerations drove the choice of these two studies: (i) they were the first to study the “moving rubber hand illusion”; (ii) they contrasted synchronous and asynchronous conditions; and (iii) they used the same proprioceptive drift task that we decided to use. However, because our main independent variable in this experiment involved mental imagery and experiments investigating the cross-modal influence of imagined sensory stimuli on real sensory perception had weaker effect sizes in some of our earlier experiments (Berger and Ehrsson 2013), we opted to increase the sample size by 20% (versus Kalckert and Ehrsson 2012) to allow for greater individual variability and expected attrition. All participants were recruited from the student population in the Stockholm area, were naïve to the purpose of the experiment (i.e., participants were screened for their exposure to body ownership illusions in other experiments in the laboratory, and the purpose of the experiment was not revealed to participants until the conclusion of the experiment), were healthy, reported no history of psychiatric illness or neurologic disorder, and reported no impairments of hearing or vision (or had corrected-to-normal vision). Data collection was stopped once the appropriate number of participants was reached, and the experiment was fully counterbalanced. All participants provided written informed consent before the start of the experiment, and the Swedish Ethical Review Authority approved the experiments.

### Materials and procedures

**Fig. 1:**
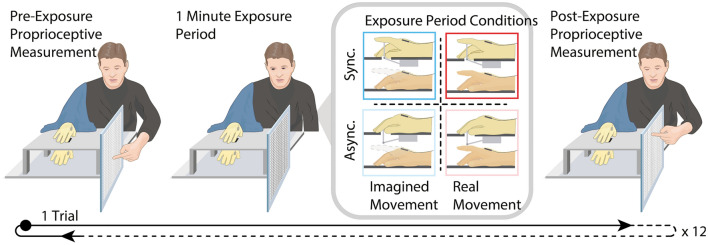
Experimental Setup and Design. Example timeline of a given trial and overview of the experimental conditions. The participants performed pre- and post-exposure proprioceptive estimations of the perceived location of their real right hand. For the exposure periods, the participants either imagined moving their index finger while a robotic hand’s index finger was moving synchronously or asynchronously or actually moved their index synchronously or asynchronously with the finger movements of the robotic hand in a 2 × 2 experimental design. The participants provided their responses to the ownership and agency questionnaires following each trial. Each exposure condition was repeated three times in four randomized blocks, resulting in 12 total trials

Each participant was seated with their right hand placed inside a box (305 mm × 203 mm × 127 mm) on the table in front of them (see Fig. 1). An EMG electrode was placed on the participant’s first dorsal interosseous (FDI) muscle, and a latex glove was placed on the participant’s hand (the glove covered the EMG electrode as well). The robotic hand was on top of the box in clear view of the participant and directly over the participant’s real hand. For consistency, a ‘dummy’ EMG electrode was also attached to the robotic hand in the approximate anatomical location of the FDI muscle, and a latex glove was placed on the artificial hand over the dummy electrode. A cloth was draped between the right shoulder of the participant and the most proximal third of the dorsal portion of the robotic hand so that the participant could not see their real arm or their real hand placed inside the box.

Before starting the experiment, the participants were given instructions regarding how to imagine the finger movement. In this short training session, the participants were instructed to imagine moving their index finger up and down at a frequency of 1 Hz, and they mentally rehearsed this action. The participants were instructed to use kinesthetic-motor imagery, i.e., imaging the somatosensory and motoric feeling of actively moving their finger from a first-person perspective (Jeannerod and Decety 1995).

In addition, immediately before starting each 1-min exposure period in the experiment, there was a verbal countdown from 5 (played through a loudspeaker and controlled by a script) at a regular rhythm of 1 Hz that provided the base frequency at which to imagine (or move) the finger. The participants started to imagine (or move) the index finger movement in phase with these countdown cues when the robot was still immobile. Then, at the end of the countdown, the robot “took over” and started to move its finger either at the same frequency (synchronous condition) as the preceding verbal countdown or with a delay of 500 ms (asynchronous condition). Simultaneously, the participants kept imagining or executing the movements at the original rhythm, that is, either synchronously or asynchronously with the robot. The participants were not explicitly instructed to imagine or execute movements synchronously or asynchronously but simply to maintain the regular rhythm of the imagined or executed finger movements irrespective of the robot hand’s finger movements. Thus, the participants received the same instructions in the synchronous and asynchronous conditions.

The movement of the index finger of the robotic hand was controlled using a servo motor mounted to the underside of the top surface of the box on top of which was the robotic hand and was driven by custom software written in C +  + that was run on a Linux computer (Ubuntu 16.04). A small metal bar connected the bottom of the index finger of the robotic hand to the servo motor through a small hole in the top surface of the box. A wave function was used to create a naturalistic upward and then downward movement of the right index finger. The onset of the movement of the robotic hand was synchronized with the instructed onset of the imagined or real finger movement of the participants in the synchronous imagery and real motor movement conditions and was delayed by 500 ms in the asynchronous imagery and real motor movement conditions. The EMG recording of the right FDI was triggered simultaneously with the onset of the (instructed) imagined finger movement or the real motor movement in both the synchronous and asynchronous conditions. The participants continued imagining the movement or moving their finger up and down at the same frequency (60 bpm, based on the paced countdown anteceding the synchronous or asynchronous movement of the robotic hand) for a period lasting 1 min, while the index finger of the robotic hand moved at the same frequency and the same duration. Auditory cues were used to instruct the participants when to start or stop the imagined or real finger movement. The participants wore headphones playing white noise (60 dB) during the experiment to block out the sound of the robotic hand motor.

Prior to the start of and just following each 1-min exposure period (i.e., when the participants were imagining the movement of their right index finger and the robotic hand was moving synchronously or asynchronously), the participants were instructed to close their eyes and to indicate the perceived position of their right index finger. Consistent with previous studies (Kalckert and Ehrsson 2012, 2014), the participants were instructed to make one rapid accurate pointing movement with their left index finger along a board attached to the side of the box in which was the participants’ right hand (see Fig. 1). The performed movement was on a vertical axis, according to previous studies (Kalckert and Ehrsson 2012, 2014). The final position of each participant’s left index finger was marked with a pen on a sheet of paper attached to the board (based on the method introduced in Holmes et al. 2004). Great care was taken to mark the position corresponding to the center of the participant’s index finger at each trial. The difference between the participants’ perceived location of their right index finger before and after each exposure period served as our measure of proprioceptive drift. Each condition (i.e., Imagery Sync, Imagery Async, Real Sync, Real Async) was repeated three times, and the mean drift for each condition was compared across participants. The imagery and real conditions were split into two separate blocks that were counterbalanced across participants. The order of each condition within these blocks was random. In two planned comparisons, we examined whether the proprioceptive drift toward the robotic hand was greater in the synchronous than in the corresponding asynchronous control conditions.

Following the last repetition of each condition, the participants were asked to fill out a brief questionnaire probing their sense of ownership and their sense of agency over the robotic hand. The questionnaire consisted of eight statements. Two of the statements probed the participants’ sense of ownership over the robotic hand (e.g., “I felt as if the rubber hand were my hand”); two statements served as control statements intended to control for compliancy (e.g., “I felt as if my (real) hand were turning rubbery”) (see Table 1 for the full list of statements); two statements probed the participants’ sense of agency over the robotic hand (e.g., “I felt as I could control the movements of the rubber hand”); and two statements served as sense of agency-related control statements (e.g., “I felt as if the rubber hand were  controlling me”). The participants responded on a scale from − 3 (strongly disagree) to 3 (strongly agree) to indicate their level of agreement with each statement on the questionnaire.

**Table 1:** All questions were delivered through the questionnaire after each condition

Question type	Statement
Ownership	I felt as if the rubber hand were my hand
Ownership	I felt as if I was looking at my own hand
Ownership control	I felt as if my (real) hand were turning “rubbery”
Ownership control	I felt as if I no longer had a right hand; as if my right hand had disappeared
Agency	I felt as if I could control the movements of the rubber hand
Agency	The rubber hand moved like I wanted it to, as if it were obeying my will
Agency control	I felt as if the rubber hand were controlling me
Agency control	I felt as if the rubber hand had a will of its own

### Data analysis

To analyze the results of the questionnaire, the responses to the two ownership questions were averaged together, as were the responses to the ownership control, agency, and agency control questions. Consistent with previous work (Kalckert and Ehrsson 2012), the averaged responses from the ownership- and agency-related statements reflect the participants’ general subjective experience of the sense of ownership and agency and are therefore referred to as ownership and agency ratings. In line with our a priori hypotheses, we planned four comparisons: Imagery Sync versus Imagery Async for ownership, Real Sync versus Real Async for ownership, Imagery Sync versus Imagery Async for agency, and Real Sync versus Real Async for agency.

To further investigate the questionnaire data, we used post hoc comparisons corrected for multiple comparisons using the Bonferroni‒Holm method (*N* = 10 post hoc comparisons). Shapiro‒Wilk tests were first conducted to verify that the data were normally distributed. Wilcoxon signed-rank tests were used when the paired differences failed to meet the assumption of normality (Shapiro‒Wilk < 0.05), whereas *t* tests were used when normality was met (Shapiro‒Wilk > 0.05). We reported effect sizes depending on what test was used, Cohen’s *d*_z_ when parametric tests were run (Lakens 2013), and the matched-pairs rank biserial correlation (*r*_C_) when nonparametric tests were used (Kerby 2014; King et al. 2018).

In the results section, we first report the main analysis of the entire sample (*N* = 24), which was planned before the data collection and conducted to test our hypotheses; second, we report the post hoc analysis for the responder group (*N* = 14), which was done to corroborate our main findings. In this latter analysis, we defined as “responders” those participants who answered the ownership statements affirmatively in the real synchronous condition (the average between the two ownership statements scores should be bigger than 0, i.e., mean ownership rating > 0). Proprioceptive drift data were analyzed with the same statistical methods as those used for the questionnaire data.

All analyses and statistical tests were performed using the statistical software R (R Core Team 2021).

### Results from the entire sample (*N* = 24)

#### Ownership ratings

**Fig. 2:**
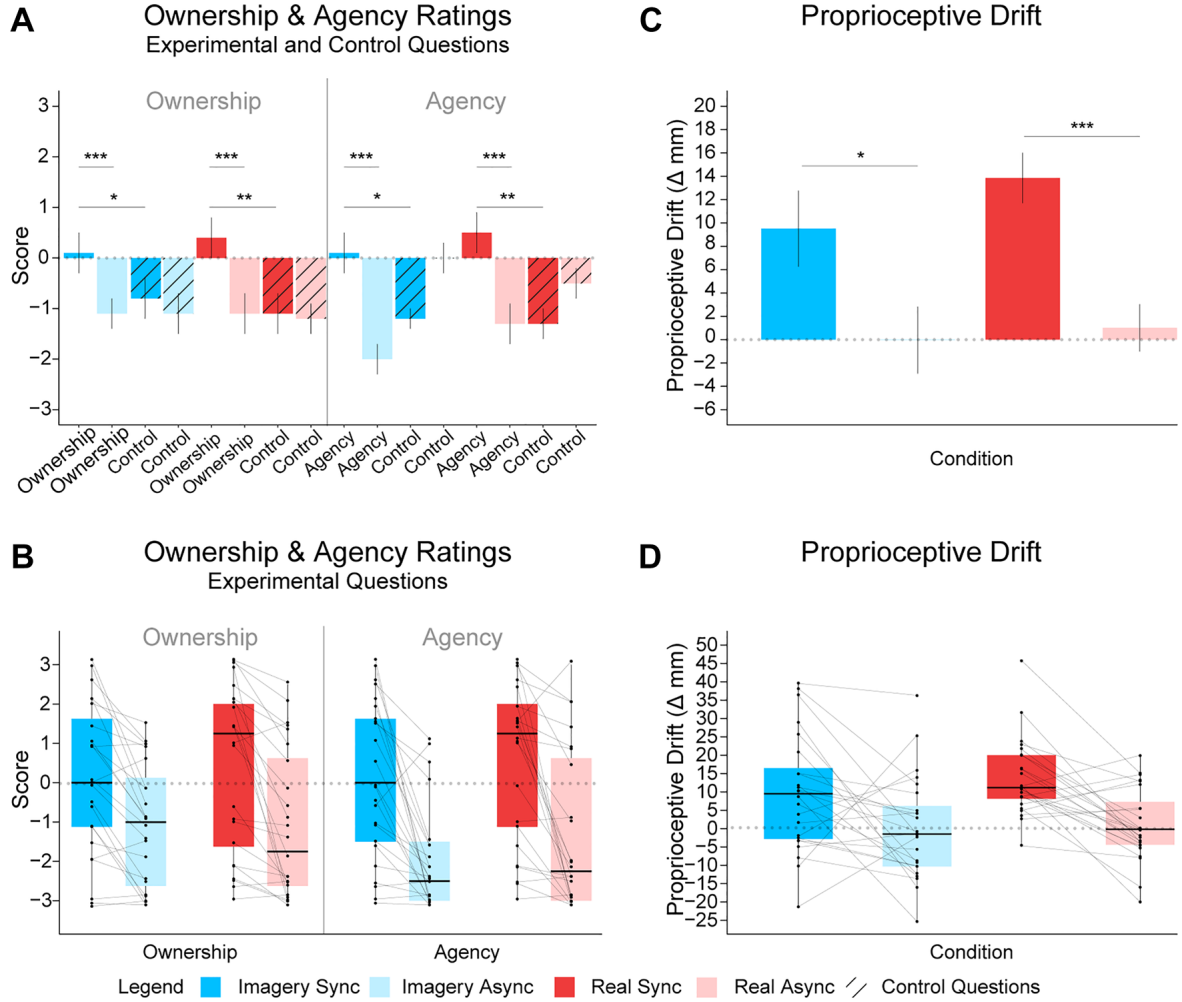
Subjective and Indirect Behavioral Measures of the Moving Rubber Hand Illusion Induced by Motor Imagery or Real Movement.** A** Mean ratings for the sense of ownership and the sense of agency for each condition collapsed across the question type (i.e., experimental or control). **B** Boxplots showing individual data points and paired lines for planned comparisons of ownership and agency ratings between the synchronous and asynchronous conditions only for the pooled experimental questions. **C** The mean difference between the participants’ perceived location of their real hand for each condition. **D** Boxplots showing individual data points and paired lines for planned comparisons in the proprioceptive drift localization task. Asterisks between bars indicate significant differences between synchronous and asynchronous conditions in the motor imagery and real motor movement conditions. Asterisks between bars in a. and b. indicate significant differences between key comparisons (*p < 0.05, **p < 0.01, ***p < 0.001), and error bars represent ± SE. Legend: Dark blue represents the Imagery Synchronous condition, light blue represents the Imagery Asynchronous condition, dark red represents the Real Synchronous condition, and light red represents the Real Asynchronous condition. Additionally, the striped pattern is used to differentiate the control from the experimental questions

Planned comparisons revealed a significant difference between the ownership ratings in the Imagery Sync condition (*M* = 0.10, SD =  ± 1.89) and those in the Imagery Async condition (*M* =  − 1.10, SD =  ± 1.57) (see descriptive statistics in Table S1 in the Supplementary Information) (*t*_23_ = 5.144, *p* < 0.0001, 95% CI [0.722, 1.694], *d*_z_ = 1.05) (see Fig. 2a and b) and a significant difference between ownership ratings in the Real Sync condition (*M* = 0.44, *SD* =  ± 2.11) and those in the Real Async condition (*M* =  − 1.08, *SD* =  ± 1.91) (*V*_23_ = 227, *p* < 0.001, 95% CI [0.750, 2.500], *r*_C_ = 0.965).

Post hoc comparisons revealed a significant difference between ownership ratings and ownership control ratings in the Imagery Sync condition (*M* =  − 0.83, *SD* =  ± 1.84) (*t*_23_ = 2.453, *p* = 0.02, *p*_BH-corr_ = 0.044, *d*_z_ = 0.50, 95% CI [0.147, 1.728]). There was no significant difference between ownership ratings (*M* =  − 1.10, SD = 95% CI 1.57) and ownership control ratings (*M* =  − 1.08, SD = 95% CI 1.77) for the Imagery Async condition (*t*_23_ =  − 0.053, *p* = 0.96, 95% CI [− 0.841, 0.799], *d*_z_ =  − 0.01). There was also a significant difference between the ownership and ownership control ratings (*M* =  − 1.06, *SD* =  ± 1.87) in the Real Sync condition (*t*_23_ = 3.578, *p* = 0.002, *p*_BH-corr_ = 0.005, 95% CI [0.633, 2.367], *d*_z_ = 0.730) but no significant difference between ownership and ownership control ratings (*M* =  − 1.25, SD =  ± 1.67) in the Real Async condition (*t*_23_ = 0.569, *p* = 0.575, 95% CI [− 0.439, 0.772], *d*_z_ = 0.116). Furthermore, comparing the participants’ sense of ownership in the Imagery Sync and Real Sync conditions revealed that there was no significant difference in the sense of ownership in these conditions (*V*_23_ = 97.5, *p* = 0.79, *r*_C_ =  − 0.07, 95% CI [− 1.25, 0.75]; see Fig. 2a and b).

#### Agency ratings

A planned comparison between the agency ratings for the Imagery Sync (*M* = 0.08, SD =  ± 1.95) and Imagery Async conditions (*M* =  − 1.98, SD =  ± 1.31) (see descriptive statistics in Table S1 in the Supplementary Information) revealed significantly greater feelings of agency over the robotic hand in the Imagery Sync condition (*t*_23_ = 5.236, *p* < 0.001, 95% CI [1.248, 2.877], *d*_z_ = 1.069) (see Fig. 2a and 2b). Furthermore, analysis of the real motor movement conditions revealed a significant difference between the agency ratings in the Real Sync condition (*M* = 0.54, SD =  ± 1.96) and those in the Real Async (*M* =  − 1.29, SD =  ± 2.01) condition (*t*_23_ = 4.459, *p* = 0.0002, *p*_BH-corr_ = 0.0005, 95% CI [0.983, 2.684], *d*_z_ = 0.91).

**Fig. 3:**
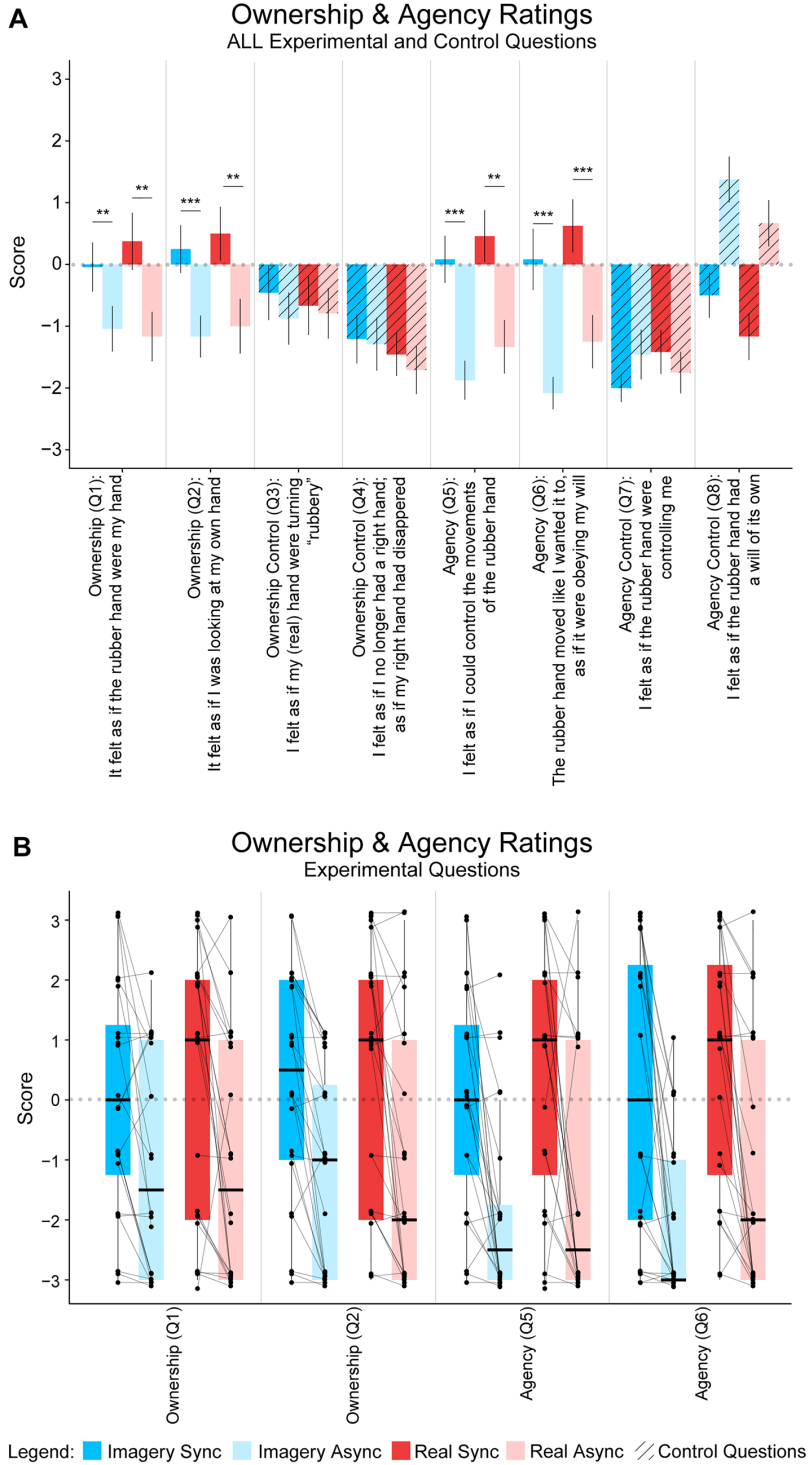
Mean Ownership and Agency Rating for All Questions. **A** The mean rating for the individual ownership, agency, and control statements for the Imagery Sync, Imagery Async, Real Sync, and Real Async conditions. Asterisks between bars indicate significant differences between key comparisons (*p < 0.05, **p < 0.01, ***p < 0.001), and error bars represent ± SE. The statistical analysis can be found in the Supplementary Information. **B** Boxplots show medians, individual data points and pairwise comparison lines for the most important comparisons. Only experimental questions related to body ownership and agency are shown. Legend: Dark blue represents the Imagery Synchronous condition, light blue represents the Imagery Asynchronous condition, dark red represents the Real Synchronous condition, and light red represents the Real Asynchronous condition. Additionally, the striped pattern is used to differentiate the control from the experimental questions

A post hoc comparison showed a significant difference between the agency ratings (*M* = 0.08, SD =  ± 1.95) and the agency control ratings (*M* =  − 1.25, SD =  ± 1.03) in the Imagery Sync condition (*t*_23_ = 2.739, *p* = 0.011, *p*_BH-corr_ = 0.029, 95% CI [0.326, 2.340], *d*_z_ = 0.559). As expected, in the Imagery Async condition, the agency ratings were not higher than the agency control ratings (*M* =  − 0.04, SD =  ± 1.52), in line with the lack of agency; in contrast, the participants disagreed significantly more with the experience of agency than with the control statements (*V*_23_ = 18, *p* < 0.001, *p*_BH-corr_ = 0.004, 95% CI [− 2.999, − 1.500], *r*_C_ =  − 0.86). This result appears to be driven by uncertainty related to the agency control statement, “I felt as if the rubber hand had a will of its own”, in the Imagery Async condition, which in hindsight is perhaps understandable given the lack of agency in this condition (*M* = 1.38, SD =  ± 1.8, see Fig. 3). There was also a significant difference between the agency ratings and the agency control ratings (*M* =  − 1.29, SD =  ± 1.36) in the Real Sync condition (*t*_23_ = 3.643, *p* = 0.0014, *p*_BH-corr_ = 0.005, 95% CI [0.792, 2.875], *d*_z_ = 0.744). As in the above-described imagery condition, in the Real Async condition, participants disagreed more with statements probing their sense of agency than the control statements (*M* =  − 0.54, SD =  ± 1.30) (although this difference was just below the threshold for statistical significance, *t*_23_ =  − 2.015, *p* = 0.056, 95% CI [− 1.52, 0.020], *d*_z_ =  − 0.411), in line with lack of agency in the former condition. Finally, comparing the agency ratings between Imagery Sync and Real Sync did not reveal any significant difference (*t*_23_ =  − 1.3792, *p* = 0.18, 95% CI [− 1.1458; 0.229], *d*_z_ = 0.00).

#### Complementary analysis

We ran an additional analysis to further corroborate our questionnaire findings and control for possible unspecific effects related to suggestibility. To this end, we subtracted the ownership control score (or the agency control score) from the ownership score (or the agency score) and used those difference values (‘control adjusted ownership score’ and ‘control adjusted agency score’, respectively) in our comparisons between the synchronous and asynchronous conditions (see Fig. S1 in the Supplementary Information). For the control-adjusted agency score, there was a significant difference between Real Sync and Real Async (*t*_23_ = 5.581, *t*_23_ < 0.001, BF_10_ > 100, *d*_z_ = 1.139) as well as between Imagery Sync and Imagery Async (*t*_23_ = 5.078, *t*_23_ < 0.001, BF_10_ > 100, *d*_z_ = 1.037). For the control-adjusted ownership score, there was a significant difference between Real Sync and Real Async (*V* = 191.5, *p* = 0.001, BF_10_ = 34.658, *r*_C_ = 0.824) as well as between Imagery Sync and Imagery Async (*V* = 183.5, *p* = 0.003, BF_10_ = 14.122, *r*_C_ = 0.748). Thus, these findings corroborate the results from the main questionnaire analyses described above.

#### Proprioceptive drift

Planned comparisons showed a significant shift (in millimeters) toward the robotic hand in the Imagery Sync (*M* = 9.5, SD =  ± 16.0) condition compared with the Imagery Async (*M* =  − 0.0, SD =  ± 14.1) condition (*t*_23_ = 2.38, *p* = 0*.*025, 95% CI [1.250, 17.860], *d*_z_ = 0.486; see Fig. 2c and d). Similarly, a significant difference in the perceived location of the participants’ real hand toward the robotic hand was observed between the Real Sync (*M* = 13.861, SD =  ± 10.549) and Real Async conditions (*M* = 1.0, SD =  ± 10.0) (*t*_23_ = 4.83, *p* < 0*.*001, 95% CI [7.347, 18.347], *d*_z_ = 0.986).

A post hoc paired *t* test comparing the participants’ proprioceptive drift in the Imagery Sync and Real Sync conditions revealed that there was no significant difference in proprioceptive drift in these conditions (*t*_23_ =  − 1.16, *p* = 0.256, *d*_z_ =  − 0.24, 95% CI [− 12.064, 3.370]).

#### Correlation between subjective and indirect behavioral measures of ownership

**Fig. 4:**
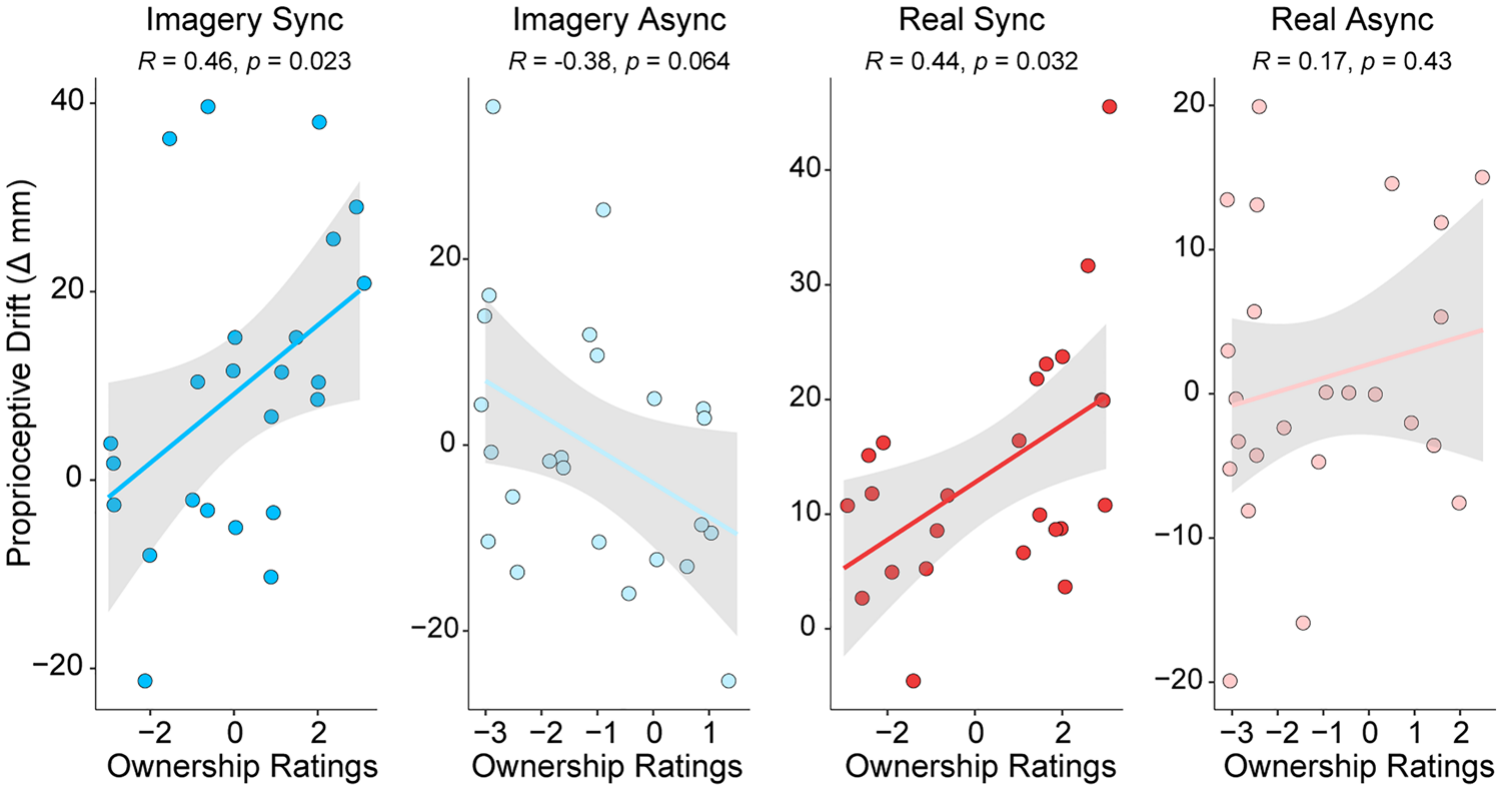
Correlation plots. Regression plots and 95% confidence interval bands showing the significant positive relationship between the sense of ownership and proprioceptive drift in the Imagery Sync and Real Sync conditions and a negative trend or no relationship in the Imagery Async and Real Async conditions, respectively. Legend: Dark blue represents the Imagery Synchronous condition, light blue represents the Imagery Asynchronous condition, dark red represents the Real Synchronous condition, and light red represents the Real Asynchronous condition

An examination of the relationship between proprioceptive drift and the sense of ownership in the Imagery Sync condition revealed a significant positive relationship between drift in the perceived location of one’s hand and participants’ sense of ownership over the robotic hand (*r*_s (1,22)_ = 0.46, *p* = 0.023, 95% CI [0.051, 0.726]). That is, the further the shift in participants’ perceived location of their real hand toward the robotic moving hand in the Imagery Sync condition, the stronger they reported feeling a sense of ownership over the robotic hand (see Fig. 4). There was also a significant positive relationship between proprioceptive drift and the sense of ownership in the Real Sync condition (*r*_s (1,22)_ = 0.438, *p* = 0.03, 95% CI [0.043, 0.719]; see Fig. 4). A negative trend was observed for the relationship between proprioceptive drift and the sense of ownership (i.e., the weaker the confidence was in rejecting the sense of ownership, the smaller the drift in hand position sense toward the robotic hand) in the Imagery Async condition (*r*_s(1,22)_ =  − 0.384, *p* = 0.064, 95% CI [− 0.691, 0.035]). There was no significant relationship between proprioceptive drift and the sense of ownership in the Real Async condition (*r*_s (1,22)_ = 0.17, *p* = 0.427, 95% CI [− 0.263, 0.57]).

#### Finger movement analysis

**Fig. 5:**
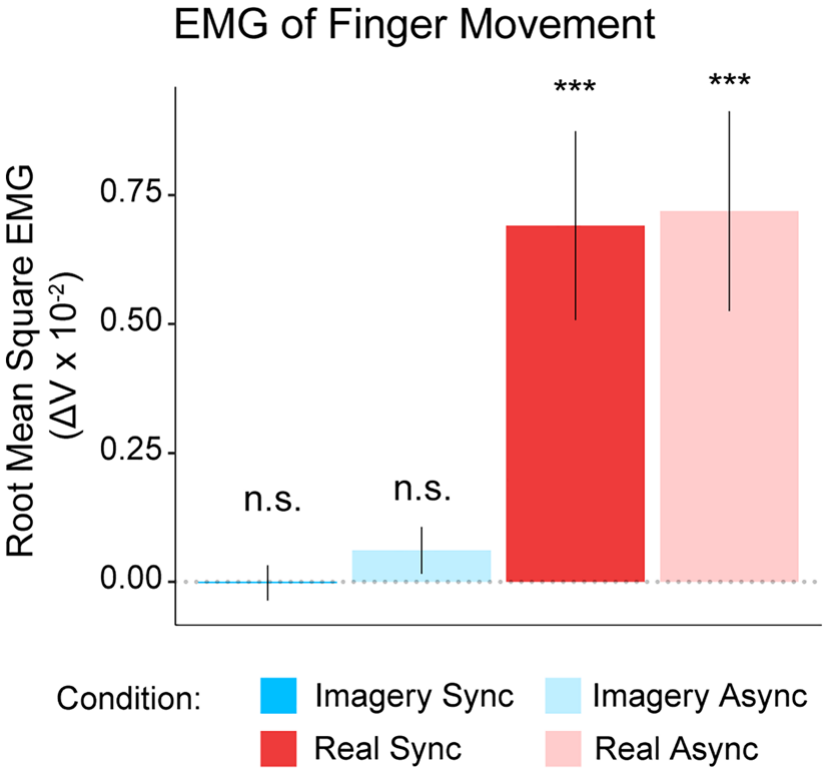
EMG Measurement of Participants’ Real Hand. Root mean square of the change in the EMG response (versus baseline) during the Imagery Sync and Imagery Async conditions and the Real Sync and Real Async conditions. Asterisks (***p ≤ 0.001) above the bars indicate significant differences in one-sample comparisons against a test value of 0. Labels reading “n.s.” above bars indicate no significant difference (*p* > 0.05) for one-sample comparisons. Legend: Dark blue represents the Imagery Synchronous condition, light blue represents the Imagery Asynchronous condition, dark red represents the Real Synchronous condition, and light red represents the Real Asynchronous condition

To verify whether the participants followed the motor imagery and motor movement instructions during the experiment, the root mean square of the EMG signal from the participants’ right FDI muscle from a 1-min baseline recording was subtracted from the root mean square (RMS) EMG signal from each condition. One-sample *t* tests against a test value of 0 revealed that there was no significant muscular contraction during the Imagery Sync condition (*t*_23_ = 0.12, *p* = 0.91, *d*_z_ = 0.024, 95% CI [− 0.001, 0.001]) or during the Imagery Async condition (*t*_23_ = 1.39, *p* = 0.18, *d*_z_ = 0.028, 95% CI [− 0.000, 0.002]; see Fig. 5). A further paired-samples *t* test showed no significant differences in EMG signals between the Imagery Sync and Imagery Async conditions (*t*_23_ =  − 1.50, *p* = 0.15, *d*_z_ = 0.30, 95% CI [− 0.001, 0.000]). In addition, we conducted a Bayes factors analysis on the RMS of the EMG signal. This was conducted utilizing the default priors (R package “BayesFactor” by (Morey et al. 2015). Bayes factors in favor of the alternative hypothesis (i.e., *H*_1_; *μ* ≠ 0) compared to the null hypothesis (i.e., *H*_0_; *μ* = 0) are reported as “BF_10_” (i.e., ${BF}_{10}=\frac{P(D|{H}_{1})}{P(D|{H}_{0})}$), whereas Bayes factors in favor of the null hypothesis compared to the alternative hypothesis are reported as BF_01_. This analysis revealed BF_10_ values of 0.22 and 0.5 in the Imagery Sync and Imagery Async conditions, respectively, suggesting substantial and anecdotal evidence (Jefferys 1961) in favor of the null hypothesis for these comparisons. A BF test comparing the mean difference between Imagery Sync and Imagery Async conditions also revealed anecdotal evidence (BF_01_ = 1.754) in favor of the null hypothesis (i.e., *μ*_Imagery Sync_ − *μ*_Imagery Async_ = 0). Together, these results rule out the possibility that small muscular contractions of movement could explain the stronger rubber hand illusion in the Imagery Sync than in the Imagery Async condition (for further control analyses, see the Results section in the Supplementary Information). Regarding the real movement conditions, one-sample *t* tests revealed significant EMG activity in the Real Sync (*t*_23_ = 3.71, *p* = 0.001, *d*_z_ = 0.758, 95% CI [0.003, 0.011]) and Real Async (*t*_23_ = 3.62, *p* = 0.001, *d*_z_ = 0.739, 95% CI [0.003, 0.011]) conditions, as expected, and a BF test revealed very strong evidence in favor of the alternative hypothesis (i.e., *μ* ≠ 0) in the Real Sync (BF_10_ = 31.06) and strong evidence in favor of the alternative hypothesis (*μ* = 0) in the Real Async condition (BF_10_ = 25.72) (see Supplementary Information for additional correlational analyses).

### Results from the responder sample (*N* = 14)

**Fig. 6:**
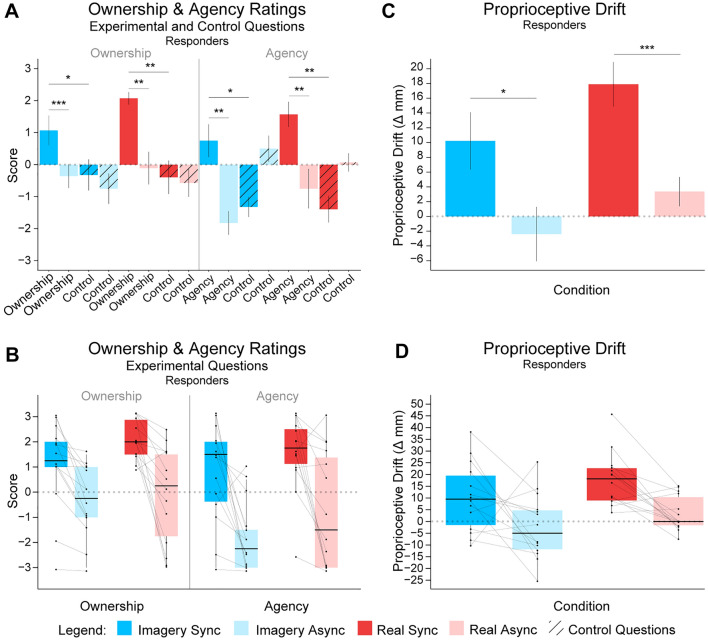
Mean Ownership and Agency Ratings and Proprioceptive Drift in the Illusion Responder Group (*N* = 14).** A** The mean ratings for the individual ownership, agency, and control statements for the Imagery Sync, Imagery Async, Real Sync, and Real Async conditions in the responder group (*N* = 14). The responder group included the participants who affirmed the ownership statements in the real synchronous condition with a mean rating > 0. **B** Boxplots show medians, individual data points and pairwise comparison lines between key comparisons. Only the pooled experimental questions are shown. **C** The proprioceptive drift toward the rubber hand was larger in the Imagery Sync condition than in the Imagery Async condition and higher in the Real Sync condition than in the Real Async condition. **D** Boxplots, individual data points and pairwise comparison lines are shown for the most important comparisons. Asterisks between bars in a. and b. indicate non-corrected significant differences between comparisons (*p < 0.05, **p < 0.01, ***p < 0.001), and error bars represent ± SE. Legend: Dark blue represents the Imagery Synchronous condition, light blue represents the Imagery Asynchronous condition, dark red represents the Real Synchronous condition, and light red represents the Real Asynchronous condition

Since the responder group analysis was performed post hoc and not planned a priori, the statistical significance was corrected by the Bonferroni‒Holm method for multiple comparisons (comparisons = 10) and is reported in this paragraph. Ratings in the ownership statement were significantly higher in Imagery Sync than in Imagery Async (*t*_13_ = 4.684*, p* = 0.0004, *p*_BH*-*corr_ = 0.002, 95% CI [0.7697; 2.08745], *d*_*z*_ = 1.25) and in Real Sync than in Real Async (*t*_13_ = 4.367*, p* = 0.0008, *p*_BH*-*corr_ = 0.002, 95% CI [1.1007; 3.2564], *d*_z_ = 1.25). The ratings in the agency statement were significantly higher in Imagery Sync than in Imagery Async (*t*_13_ = 4.24*, p* = 0.00096, *p*_BH*-*corr_ = 0.002, 95% CI [1.26; 3.88], *d*_z_ = 1.13) and in Real Sync than in Real Async (*t*_13_ = 3.735*, p* = 0.0025, *p*_BH*-*corr_ = 0.004, 95% CI [0.979; 3.664], *d*_z_ = 0.998). Further analysis revealed that for synchronous conditions (i.e., Imagery Sync and Real Sync), the experimental ratings were higher than the control ratings (Imagery Sync, ownership: *t*_13_ = 2.424*, p* = 0.03, *p*_BH*-*corr_ = 0.038, 95% CI [0.151; 2.634], *d*_z_ = 0.648; Imagery Sync, agency: *t*_13_ = 2.8167*, p* = 0.015, *p*_BH*-*corr_ = 0.02, 95% CI [0.483; 3.66], *d*_z_ = 0.753; Real Sync, ownership: *t*_13_ = 4.433*, p* = 0.0007, *p*_BH*-*corr_ = 0.002, 95% CI [1.263; 3.665], *d*_*z*_ = 1.185; Real Sync, agency: *t*_13_ = 4.738*, p* = 0.0004, *p*_BH*-*corr_ = 0.002, 95% CI [1.665]; *d*_*z*_ = 1.267). Furthermore, the ownership statement did not differ between Imagery Sync and Real Sync (*t* =  − 2.1199*, p* = 0.054, 95% CI [− 2.0191; 0.0191], *d*_*z*_ =  − 0.566), nor did the agency statement in the same comparison (*V* =  − 2.5*, p* = 0.083, 95% CI [− 2.5, 0.5], *d*_z_ =  − 0.67). The proprioceptive drift toward the rubber hand was larger in the Imagery Sync condition than in the Imagery Async condition (*t* = 2.218*, p* = 0.0449, 95% CI [0.329; 24.909], *d*_z_ = 0.593) and significantly higher in the Real Sync condition than in the Real Async condition (*t* = 4.28*, p* = 0.0009, 95% CI [7.192, 21.855], *d*_z_ = 1.144). The results from the responder group analysis are shown in Fig. 6.

## Discussion

In this study, we investigated whether mental motor imagery (Jeannerod and Decety 1995) can elicit the moving rubber hand illusion by “replacing” real somatosensory feedback and motor commands with kinesthetic-motor imagery. The results indicated that imagining voluntary finger movements synchronously with the finger movements of a robotic hand led to a significant increase in the subjective feelings of ownership and the sense of agency over the robotic hand coupled with a significant change in the perceived location of one’s hand toward the location of the rubber hand (i.e., proprioceptive drift) compared with asynchronously imagined movements. In addition, we observed that participants who gave positive affirmative ratings of illusory ownership in the real synchronous movement condition, i.e., participants who were susceptible to the moving rubber hand illusion, also reported positive affirmative ownership illusion ratings in the synchronous motor imagery condition. In addition, EMG recordings ruled out that tiny movements or muscular contractions in the real finger during the motor imagery conditions could explain our findings. Collectively, these results suggest that mental imagery can influence the multisensory perception of one’s own body and trigger or modulate the moving rubber hand illusion, but only when the imagined movements match the veridical visual information. This finding has important implications for theories of the bodily self, the relationships between mental imagery and veridical perception, and the embodiment of BCI-controlled technology, including advanced prosthetic limbs.

The present results indicate that the functional overlap between mental imagery and sensory perception goes beyond within-modality similarities and influences on sensory perception (Kosslyn et al. 2001) and cross-modality effects in audiovisual perception (see introduction; Berger and Ehrsson 2013, 2016, 2017, 2018) as demonstrated by previous work. Here, we show that our mental simulation of action and its associated somatic sensations are capable of integrating with ongoing perception in vision (and proprioception) to alter one of the most fundamental aspects of our perceptual experience—our sense of bodily self. The present bodily illusion is elicited as a result of spatiotemporally matching visual impressions from robotic hand’s index finger movements, in view, and the imagined somatosensory (i.e., kinesthetic) sensations associated with kinesthetic-motor imagery of index finger movements (of the real hand, hidden from view). The voluntary motor commands and efference copy associated with the motor imagery (Jeannerod and Decety 1995; Kilteni et al. 2018) probably do not have a major role in the elicitation of the moving rubber hand illusion per se, as passive movement can readily trigger this illusion (Kalckert and Ehrsson 2012, 2014); instead, the internal simulation of voluntary motor commands probably has an important role in the sense of agency, as we will discuss further below. Although we know from earlier work that kinesthetic-motor imagery can influence concurrent perception of limb movement (Naito et al. 2002; Thyrion and Roll 2009) and self-touch (Kilteni et al. 2018), the present findings are important because they indicate that internally generated signals related to imagined movement sensations are sufficiently strong, well timed and rich in information to integrate with congruent veridical sensory signals from a different sensory modality (vision) and elicit a multisensory bodily illusion. From a theoretical perspective, this conclusion is interesting for several reasons. First, it extends previous work regarding the effects of imagery on audiovisual illusions to the case of a visuo-kinesthetic bodily illusion, which strengthens the support for the theory that mental imagery can integrate with sensory signals in other sensory modalities and trigger multisensory illusions. Second, our findings suggest a top-down cognitive mechanism whereby voluntary imagination can influence bodily awareness and cause changes in the sense of body ownership. Third, they provide support for perceptual accounts of mental imagery, which, although widely accepted in the field of visual imagery research (Pearson 2019; Pearson and Kosslyn 2015), is less strongly supported in multisensory and body representation research.

As said, the present study investigated how kinesthetic-motor imagery influences the sense of body ownership in the rubber hand illusion, but noteworthy, a previous study examined the opposite pattern of influence, namely, whether the rubber hand illusion can influence mental motor imagery (Ionta et al. 2013). In this study, the visuo-tactile rubber hand illusion was used to change the perceived posture of a person’s hand. This manipulation influenced mental hand rotation reaction times (Parsons 1987; an indirect behavioral index of motor imagery) in a similar way as if the real hand’s posture had changed (Ionta et al. 2013). Thus, it seems as if illusory own-body perception can influence our bodily imagination and our bodily imagination can influence how we perceive our body.

The current finding that a sense of agency over the rubber hand’s movements could also be elicited by synchronous motor imagery and visual feedback is also interesting. The sense of agency requires both voluntary motor intentions and a match between the expected sensory consequences of movement and the afferent sensory feedback of actual movements (Frith et al. 2000; Haggard 2017). The fact that agency sensations were evoked is thus in line with the notion that motor imagery involves internal simulation of voluntary motor programs and the expected sensory consequences of the imagined movements. The former, in which kinesthetic motor imagery involves internal simulation of motor programs and central motor commands, is well established (Jeannerod 1997; Jeannerod and Decety 1995; Roland et al. 1980), but the latter is much less studied. Kilteni and Ehrsson (2018) used kinesthetic-motor imagery to show that imagined self-generated touch produces an attenuation of real tactile sensations, which suggests that that type of motor imagery involves predicting the sensory consequences of the imagined movement. The present agency findings can be thought of as supporting the same idea because if the expected sensory consequences of the imagined finger movements were not predicted, the agency feeling would probably be weaker (or even absent). Thus, in the current synchronous motor imagery condition, the visual feedback matches the internally generated sensory predictions from the imagined movements, which contributes to the agency sensations to arise. That motor imagery coupled with arbitrarily sensory feedback that matches learned sensorimotor associations can lead to a sense of agency is supported by BCI research (Braun et al. 2016; Nierula et al. 2021), presumably reflecting the flexibility with which the mind can link intentions with outcomes through learning, internal models and cognitive postdictive processes (Caspar et al. 2015; Synofzik et al. 2008, 2013). However, the current study suggests that motor imagery can elicit agency over a moving limb that feels like one’s own, which presumably taps into the most basic form of agency that involves the sense of control of one’s own bodily movement based on sensorimotor processes (Abdulkarim et al. 2023; Farrer et al. 2013; Frith et al. 2000).

From an applied neuroscience and neuroengineering perspective, the current findings provide valuable support for the idea that motor imagery can be used to elicit illusory sensations of body ownership and agency in BCI control over virtual and robotic limbs, as suggested by earlier studies (Alimardani et al. 2013, 2016; Braun et al. 2016; Perez-Marcos et al. 2009; see Colucci et al. 2022 for a review on exoskeleton applications). Specifically, our findings add to this literature by demonstrating significant differences in the experience of ownership over a robotic rubber hand between synchronous and asynchronous kinesthetic motor imagery and visual feedback conditions and by providing evidence for significant proprioceptive drift effects in line with the classic rubber hand illusion literature (Botvinick and Cohen 1998; Kalckert and Ehrsson 2012; Tsakiris and Haggard 2005). Thus, our findings represent a step forward toward supporting the feasibility of eliciting body illusions by combining motor imagery and veridical sensory feedback. Future studies could extend the current setup so that the movements of the robotic index finger are controlled directly by brain signals using BCI technology and investigate the imagery-induced illusion effects on other effectors, such as legs (Crea et al. 2015), whole arms (Fang et al. 2019), and even entire bodies (Maselli and Slater 2013; Petkova and Ehrsson 2008). In principle, this may allow paralyzed individuals to genuinely perceive virtual and robotic limbs as part of their own body in advanced BCI applications beyond the sense of agency and the weaker feelings of embodiment that are often reported in current applications (Hochberg et al. 2006; Serino et al. 2022).

Four limitations of the current study should be discussed. First, we did not have an objective test for motor imagery performance but relied on the participants following the instructions, which is in line with many previous motor imagery studies (e.g., Ehrsson et al. 2003) but is still a limitation. However, the mental act of imagining simple repetitive finger movements is relatively easy for most people (Ehrsson et al. 2003; Sirigu et al. 1996), and the significant questionnaire and the proprioceptive drift effects we found are consistent with the notion that the majority of the participants performed the kinesthetic-motor imagery as instructed. Second, we did not assess the participants’ individual skills in performing vivid motor imagery (e.g., using validated questionnaire scales, e.g., Marks 1973, 1995). Thus, we could not examine whether individual differences in the vividness of motor imagery were related to the strength of the imagery-induced illusion. Third, the moving rubber hand illusion in the current experiment appears to be weaker (ownership rating + 0.44 in the real synchronous condition) than in the previous studies (in the range from + 1 to + 2) (Abdulkarim et al. 2023; Caspar et al. 2015; Kalckert and Ehrsson 2012, 2014). This difference could relate to fewer individuals being susceptible to the illusion in the current group due to random variation or to differences in the experimental setups and procedures, such as potentially less synchronized seen and felt finger movements between the self-paced finger movements and the robot’s steady rhythm compared with Kalckert’s mechanical connection between the two fingers that ensured near-perfect synchrony throughout the trials. Importantly, however, the moving rubber hand illusion was almost as strong in the imagery-induced version (+ 0.104) as the one elicited by synchronous real finger movements (+ 0.44), so it was as high as reasonably could be expected given an overall somewhat weaker moving rubber hand illusion in the current setup. Additionally, note that we included all the participants in the main analysis (in line with our preplanned analysis strategy), including those who did not experience a vivid moving rubber hand illusion, which, according to Kalckert and Ehrsson 2014, represented approximately 22 to 37% of healthy participants (and 41.7% in the current sample). However, according to our hypothesis, if one does not experience the rubber hand illusion with real sensorimotor stimulation in the first place, one should not be able to perceive this bodily illusion with mental imagery either. In hindsight, performing the experiment and analysis only on participants who are susceptible to the rubber hand illusion might have been preferable, and indeed, our post hoc analysis of the data where we included only individuals who affirmed the ownership statements in the real synchronous condition (with a mean rating > 0; *N* = 14) revealed clearly affirmative ownership (mean score ≥  + 1) and agency ratings in the imagery synchronous condition (coupled with significantly higher ownership ratings in this condition than in the asynchronous imagery condition, and a significant difference in proprioceptive drift between the two imagery conditions). Thus, this suggests that a vivid moving rubber hand illusion can be triggered by kinesthetic-motor imagery, at least in the subset of participants who are susceptible to the illusion. Note that these findings cannot be explained by possible differences in trait suggestibility (Lush et al. 2020) between the illusion responders and non-responders because such individual differences can explain only a small fraction of differences in rubber hand illusion ratings between such groups (approximately 7% to 11%; see SI Discussion for details). Crucially, moreover, there is no relationship between trait suggestibility and the difference in subjective ownership ratings score between synchronous versus asynchronous conditions (Ehrsson et al. 2022; Lush et al. 2020; Slater and Ehrsson 2022) or between suggestibility and the difference in proprioceptive drift between synchronous and asynchronous conditions, which is important, as it is this type of comparison upon which we base our main conclusion.

Future studies should replicate and extend the current findings with additional experimental manipulations, including varying the spatial distance (Fang et al. 2019; Kalckert and Ehrsson 2014; Lloyd 2007) and relative orientation (Ide 2013) of the seen moving rubber hand and imagined movements, to examine the spatial rules of the imagery-induced moving rubber hand illusion. Moreover, psychophysics and modeling approaches could be used to clarify the computational basis of the imagery effect on the rubber hand illusion (Chancel et al. 2022). It would also be interesting to investigate the neural mechanisms of the imagery-induced moving rubber hand illusion to determine whether it is associated with patterns of ownership-related activation in the premotor cortex, posterior parietal cortex and cerebellum similar to those of the veridical illusion (Abdulkarim et al. 2023). The possibility that the imagery-induced version of the rubber hand illusion may engage similar frontoparietal processes related to the sense of body ownership as the veridical version of the illusion would be in line with an EEG study that found that hand mental motor imagery and illusory limb ownership have similar electrophysiological correlates (modulation of the mu band of rhythmic electrical activity) in frontoparietal regions (Evans and Blanke 2013).

In summary, the work presented herein combined two lines of research: research on the multisensory construction of bodily self-awareness (i.e., sense of ownership and agency) and on the integration of mental imagery and cross-modal sensory perception. Our findings provide evidence that motor imagery influences the multisensory and sensorimotor processes involved in the sense of body ownership and sense of agency, but only when the timing of the motor imagery obeys the temporal rules of body ownership and agency. These findings provide support for the notion that kinesthetic-motor imagery can act as a surrogate for motor movements in the moving rubber hand illusion and similar paradigms, which may be critical in future development of neuro-prosthetics (Flesher et al. 2016; Musallam et al. 2004), BCI devices (Santhanam et al. 2006; Wolpaw and Mcfarland 2004), and clinical rehabilitation efforts for neurologically impaired patients (Butler and Page 2006; Page et al. 2009).

## Supplementary Information

Below is the link to the electronic supplementary material.

Supplementary file1 (XLSX 19 KB)

Supplementary file2 (DOCX 334 KB)

## Data Availability

The source data generated and analyzed in this study are available in Excel sheets as Supplementary Data.
