# Supplementary material for: Synchronous motor imagery and visual feedback of finger movement elicit the moving rubber hand illusion, at least in illusion-susceptible individuals
Source: Exp Brain Res. 2023 Mar 16;241(4):1021–39. doi: 10.1007/s00221-023-06586-w (PMC10081980; doi:10.1007/s00221-023-06586-w)
Supplement: Supplementary file 2 — Supplementary file2 (DOCX 334 KB) [file 221_2023_6586_MOESM2_ESM.docx]

**Supplementary Information:**

**Within-Subject Variability in the Ownership, Agency, Ownership Control and Agency Control Statements.**

| **Condition** | **Question** | **Mean** | **Standard Deviation (±)** | **Standard Error (±)** | **Median (1^st^Q ~ 3^rd^Q)** |
| --- | --- | --- | --- | --- | --- |
| **Imagery**  **Sync** | Ownership | 0.104 | 1.888 | 0.385 | 0 (-1.125 ~ 1.625) |
|  | Ownership Control | -0.833 | 1.84 | 0.376 | -0.75 (-3 ~ 1) |
|  | Agency | 0.083 | 1.954 | 0.399 | 0 (-1.5 ~ 1.625) |
|  | Agency Control | -1.25 | 1.032 | 0.211 | -1 (-2 ~ 0.375) |
| **Imagery**  **Async** | Ownership | -1.104 | 1.567 | 0.320 | -1 (-2.625 ~ 0.125) |
|  | Ownership Control | -1.083 | 1.773 | 0.362 | -1 (-3 ~ 0.125) |
|  | Agency | -1.979 | 1.306 | 0.267 | -2.5 (-3 ~ -1.5) |
|  | Agency Control | -0.042 | 1.517 | 0.310 | 0 (-1 ~ 1.5) |
| **Real**  **Sync** | Ownership | 0.438 | 2.113 | 0.431 | 1.25 (-1.625 ~ 2) |
|  | Ownership Control | -1.062 | 1.867 | 0.381 | -1.25 (-3 ~ 0.5) |
|  | Agency | 0.542 | 1.961 | 0.400 | 1.25 (-1.125 ~ 2) |
|  | Agency Control | -1.292 | 1.359 | 0.277 | -1.25 (-2.625 ~ -0.375) |
| **Real**  **Async** | Ownership | -1.083 | 1.909 | 0.390 | -1.75 (-2.625 ~ 0.625) |
|  | Ownership Control | -1.25 | 1.655 | 0.338 | -1.5 (-3 ~ -0.375) |
|  | Agency | -1.292 | 2.005 | 0.409 | -2.25 (-3 ~ 0.625) |
|  | Agency Control | -0.542 | 1.301 | 0.266 | -0.5 (-1 ~ 0.125) |

***Table S1.***

*The table shows the descriptive statistics (N = 24), such as medians, means, standard deviations, standard errors and quartiles.*

***Fig. S1.***


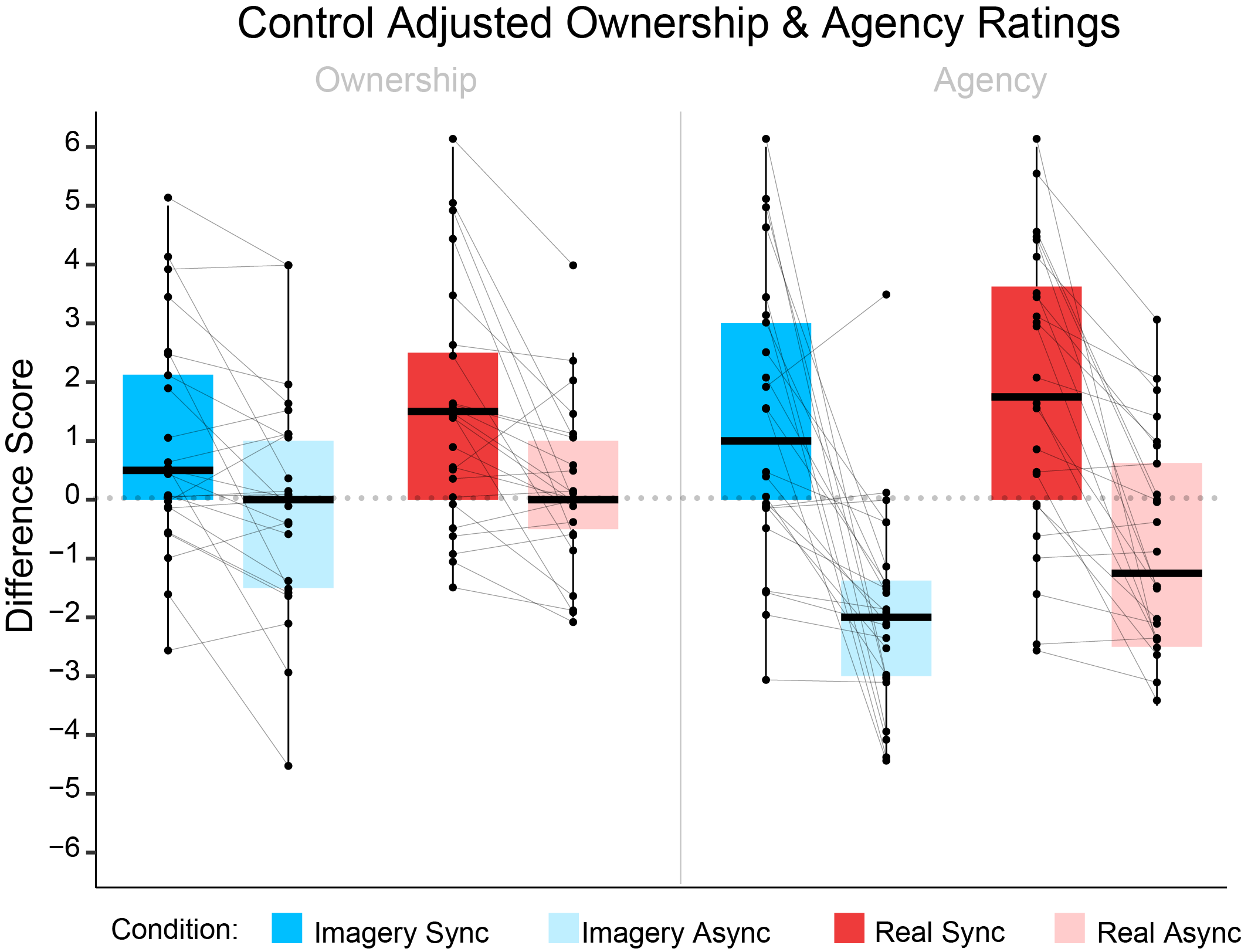
*Questionnaire results regarding ‘control adjusted’ ownership and agency statements. The figure shows boxplots, individual data points and medians.*

**SI Results**

***Analysis of Each Question of the Questionnaire.*** The analysis of each question was not planned (post hoc analysis); the statistical significance was corrected by the Bonferroni‒Holm method for multiple comparisons (comparisons = 8) and is reported in this paragraph. Ratings for question 1 (Q1) were significantly higher in Imagery Sync than in Imagery Async (V = 109.5, p = 0.005, *p*_BH-corr_ = 0.005, 95% CI [0.5, 2.5], *r_C_* = 0.825) and in Real Sync than in Real Async (t_23_ = 3.737, p = 0.001, *p*_BH-corr_ = 0.002, 95% CI [0.688, 2.395], *d_z_* = 0.763). Ratings for Q2 were significantly higher in Imagery Sync than in Imagery Async (t_23_ = 4.813, p < 0.0001, *p*_BH-corr_ = 0.0006, 95% CI [0.808, 2.026], *d_z_* = 0.982) and in Real Sync than in Real Async (V = 116, p = 0.001, *p*_BH-corr_ = 0.002, 95% CI [1, 3.5], *r_C_* = 0.933). Ratings for Q5 (Agency) were significantly higher in Imagery Sync than in Imagery Async (V = 150.5, p = 0.0005, *p*_BH-corr_ = 0.0009, 95% CI [1.5, 4], *r_C_* = 0.967) and in Real Sync than in Real Async (t_23_ = 3.415, p = 0.002, *p*_BH-corr_ = 0.003, 95% CI [0.706, 2.877], *d_z_* = 0.697). Ratings for Q6 (Agency) were significantly higher in Imagery Sync than in Imagery Async (t_23_ = 4.476, p = 0.0002, *p*_BH-corr_ = 0.0007, 95% CI [1.165, 3.168], *d_z_* = 0.914) and in Real Sync than in Real Async (V = 166.5, p = 0.0004, *p*_BH-corr_ = 0.0009, 95% CI [1, 3.5], *r_C_* = 0.947).

***Correlation Analyses between EMG and other Dependent Variables****.* Although there was no significant muscular activity in the Imagery Sync and Imagery Async conditions, indicating that participants followed instructions and only imagined the finger movements, we also examined whether there was a significant relationship between participants’ muscular activity, even if minimal, and the dependent measures of the Imagery conditions in this study. These analyses were conducted to further rule out the alternative interpretation that the drift in proprioception, sense of ownership and sense of agency experienced in the Imagery conditions of this experiment could be explained by the very subtle movements or miniscule isometric forces of the participants, rather than imagined motor movements. These analyses revealed that there was no significant relationship between participants’ EMG signal and Ownership (*r_s (1,22)_* = -0.27, *p* = .2, 95% CI: -0.61, 0.15]) or Agency ratings (*r_s (1,22)_* = -0.31, *p* = .14, 95% CI: -0.63, 0.1]) in the Imagery Sync condition. Notably, there was a significant *negative* relationship between the EMG signal and proprioceptive drift in the Imagery Sync condition, i.e., the greater the tendency for increased EMG during the experiment, the lower was the drift in the perceived location of their real hand toward the robotic hand (*r_s (1,22)_* = -0.62, *p* = .001, 95% CI: -0.82, -0.28]). BF tests also revealed anecdotal evidence in favor of the null hypothesis (i.e., *ρ* = 0) (BF_01_ *=* 1.39) for the relationship between participants’ EMG signal and the sense of ownership. Additionally, there was no evidence of a positive relationship between the EMG signal and the proprioceptive drift measurement, in line with the significant *negative* relationship revealed by the frequentist test above. These findings conclusively refute the concern that weak muscular contractions or small movements in the Imagery Sync condition could explain the proprioceptive drift in this condition. Moreover, this result may be an indication of participants’ overall task compliance with the instruction to relax their hand and fingers in the Imagery conditions. No significant correlation between muscular activity and ownership ratings (*r_s (1,22)_* = -0.03, *p* = .9, 95% CI: -0.425, 0.381]), agency ratings (*r_s (1,22)_* = -0.24, *p* = .26, 95% CI: -0.58, 0.18]), or proprioceptive drift (*r_s (1,22)_* =  -0.1, *p* = .64, 95% CI: -0.58, 0.18]) was observed for the Imagery Async condition. BF tests revealed weak evidence in favor of the null hypothesis (*ρ* = 0) for the relationship between the EMG signal in the Imagery Async condition and the sense of Ownership (BF_10_ = 0.48), agency (BF_10_ = 0.44), and proprioceptive drift (BF_10_ = 0.44).

**SI Discussion**

It is unlikely that possible differences in trait suggestibility (Lush et al., 2020) between illusion responders and illusion nonresponders can explain our findings from the responder group. This can easily be demonstrated by analyzing the data from Lush et al. (2020). If we define responders as having a mean subjective score (S1-S3) greater than to zero (the same criteria as in the current study), then the difference in mean the Sussex-Waterloo Scale of Hypnotisability (SWASH), which is a scale that measures suggestibility, between responders and nonresponders is 0.451897. According to Lush and Seth (2022), one SWASH corresponds to a 0.60 illusion rating score (Slater and Ehrsson’s estimate is approximately 0.63 (Slater & Ehrsson, 2022)), but we will use Lush’s estimate in the current example). In that case, 0.27 of the illusion rating difference between the responders and nonresponders would be explained by differences in trait suggestibility. Note that in the Real Sync condition, the difference in mean ownership rating score between the responders (+2.07) and the nonresponders (-1.85) is 3.92, and in the Imagery Sync condition, this difference is 2.31 (responders: 1.07 and nonresponders: -1.25). Thus, differences in trait suggestibility may explain only a small fraction (approximately 7% to 11%) of the difference in the rubber hand illusion between the two groups. Furthermore, note that our key statistical comparisons are between synchronous and asynchronous conditions, and such condition-specific differences show no relationship with trait suggestibility (Ehrsson et al., 2022; Slater & Ehrsson, 2022).

**References**

Ehrsson, H. H., Fotopoulou, A., Radziun, D., Longo, M. R., & Tsakiris, M. (2022). No specific relationship between hypnotic suggestibility and the rubber hand illusion. *Nature Communications*, *13*(1), 564. https://doi.org/10.1038/s41467-022-28177-z

Lush, P., Botan, V., Scott, R. B., Seth, A. K., Ward, J., & Dienes, Z. (2020). Trait phenomenological control predicts experience of mirror synaesthesia and the rubber hand illusion. *Nature Communications*, *11*(1), 4853. https://doi.org/10.1038/s41467-020-18591-6

Lush, P., & Seth, A. K. (2022). Reply to: No specific relationship between hypnotic suggestibility and the rubber hand illusion. *Nature Communications*, *13*(1), 563. https://doi.org/10.1038/s41467-022-28178-y

Slater, M., & Ehrsson, H. H. (2022). Multisensory Integration Dominates Hypnotisability and Expectations in the Rubber Hand Illusion, in press. *Frontiers in Human Neuroscience*.
